# Synthesis and in vitro and in vivo evaluation of urea-based PSMA inhibitors with increased lipophilicity

**DOI:** 10.1186/s13550-018-0440-2

**Published:** 2018-08-22

**Authors:** Martina Wirtz, Alexander Schmidt, Margret Schottelius, Stephanie Robu, Thomas Günther, Markus Schwaiger, Hans-Jürgen Wester

**Affiliations:** 1 Pharmaceutical Radiochemistry, Technische Universität München, Garching, Germany; 2 Department of Nuclear Medicine, Klinikum rechts der Isar, Technische Universität München, Munich, Germany

**Keywords:** PSMA, Prostate cancer, Radiopharmaceutical, PET, Endoradiotherapy

## Abstract

**Background:**

Several radiolabeled prostate-specific membrane antigen (PSMA) inhibitors based on the lysine-urea-glutamate (KuE) motif as the pharmacophore proved to be suitable tools for PET/SPECT imaging of the PSMA expression in prostate cancer patients. PSMA I&T, a theranostic tracer developed in our group, was optimized through alteration of the peptidic structure in order to increase the affinity to PSMA and internalization in PSMA-expressing tumor cells. However, further structural modifications held promise to improve the pharmacokinetic profile.

**Results:**

Among the investigated compounds **1**–**9**, the PSMA inhibitors **5** and **6** showed the highest PSMA affinity (lowest *IC*_*50*_ values) after the introduction of a naphthylalanine modification. The affinity was up to three times higher compared to the reference PSMA I&T. Extended aromatic systems such as the biphenylalanine residue in **4** impaired the interaction with the lipophilic binding pocket of PSMA, resulting in a tenfold lower affinity. The *IC*_*50*_ of DOTAGA-conjugated **10** was slightly increased compared to the acetylated analog; however, efficient PSMA-mediated internalization and 80% plasma protein binding of ^68^Ga-**10** resulted in effective tumor targeting and low uptake in non-target tissues of LNCaP tumor-bearing CD-1 nu/nu mice at 1 h p.i., as determined by small-animal PET imaging and biodistribution studies. For prolonged tumor retention, the plasma protein binding was increased by insertion of 4-iodo-d-phenylalanine resulting in 97% plasma protein binding and 16.1 ± 2.5% ID/g tumor uptake of ^177^Lu-**11** at 24 h p.i.

**Conclusions:**

Higher lipophilicity of the novel PSMA ligands **10** and **11** proved to be beneficial in terms of affinity and internalization and resulted in higher tumor uptake compared to the parent compound. Additional combination with para-iodo-phenylalanine in the spacer of ligand **11** elevated the plasma protein binding and enabled sustained tumor accumulation over 24 h, increasing the tumor uptake almost fourfold compared to ^177^Lu-PSMA I&T. However, high renal uptake remains a drawback and further studies are necessary to elucidate the responsible mechanism behind it.

**Electronic supplementary material:**

The online version of this article (10.1186/s13550-018-0440-2) contains supplementary material, which is available to authorized users.

## Background

Due to the high overexpression in the majority of prostate cancers and low expression density in healthy tissues, the cell surface-bound zinc metalloprotease prostate-specific membrane antigen (PSMA) has attracted attention as a target for diagnosis and therapy of prostate cancer [1–3]. Recently, targeting of PSMA has been described for a variety of small molecule inhibitors and antibodies, and (((S)-5-amino-1-carboxypentyl)carbamoyl)-l-glutamic acid (l-lysine-urea-l-glutamate; KuE) was found to be a highly potent binding motif in small molecules for the active center of PSMA [4–12]. To improve the interaction of PSMA inhibitors with the enzyme, a lipophilic binding pocket (arene-binding site) was described in a distinct distance from the active center of PSMA, which induces an up to 60-fold increase in affinity for PSMA inhibitors containing a dinitrophenyl group to bind this lipophilic pocket [13]. The PSMA inhibitor ^68^Ga-HBED-CC-Ahx-KuE (PSMA-11) was described for addressing both of these binding pockets [14]. This PSMA inhibitor successfully demonstrated the diagnostic potential of PSMA-targeted molecular imaging with PET [15, 16]. Prostate cancer and its metastases were also visualized and treated with ^68^Ga-PSMA I&T PET [17, 18] and ^177^Lu-PSMA I&T radioligand therapy [17, 19–21], respectively. Compared to phenylalanine (f) in DOTAGA-ffk(Sub-KuE), a first-generation PSMA inhibitor developed by our group [22], an increased interaction of iodo-tyrosine (I-y) in DOTAGA-(I-y)fk(Sub-KuE) (PSMA I&T—for imaging and therapy) [17] with this lipophilic pocket most likely explains the higher PSMA affinity and increased internalization of PSMA I&T into PSMA-expressing cells [17]. In addition, ^111^In-PSMA I&T was successfully utilized for intraoperative detection of lymph node metastases in the context of radio-guided surgery, which supported the development of the ^99m^Tc-based analog PSMA I&S [7, 23, 24].

**Fig. 1:**
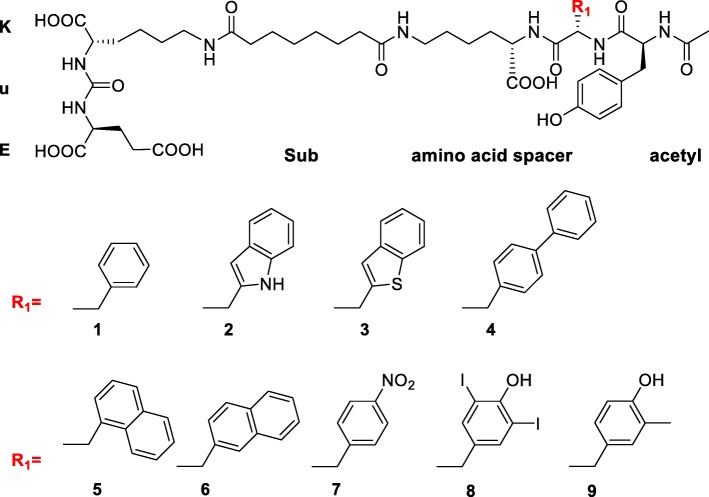
Chemical structures of the acetylated PSMA inhibitors 1–9

The objective of this work was to investigate the influence of lipophilic amino acid substitutions in the linker of a series of acetylated PSMA inhibitors (Fig. 1) based on PSMA I&T to further improve the in vivo characteristics of this class of PSMA inhibitors. Compounds **1–9** were designed to bind the active center of PSMA by interaction with the KuE motif, which was conjugated via a suberic acid spacer to a three-amino acid peptide motif (for interaction with the lipophilic binding pocket of PSMA). In this exploratory part of the study, the DOTAGA chelator (1,4,7,10-tetraazacyclododececane,1-(glutaric acid)-4,7,10-triacetic acid) in PSMA I&T was substituted by an acetyl moiety, which is entirely sufficient to disclose relevant structure-activity relationships determined by the spacer geometry. Based on the PSMA ligand Ac-Y-2-Nal-K(Sub-KuE), which revealed the highest affinity to PSMA in this series, the DOTAGA-conjugated PSMA inhibitor DOTAGA-y-2-nal-k(Sub-KuE) was synthesized from d-amino acids in the spacer to increase metabolic stability in vivo and was preclinically evaluated according to previous investigations by our group [22].

**Fig. 2:**
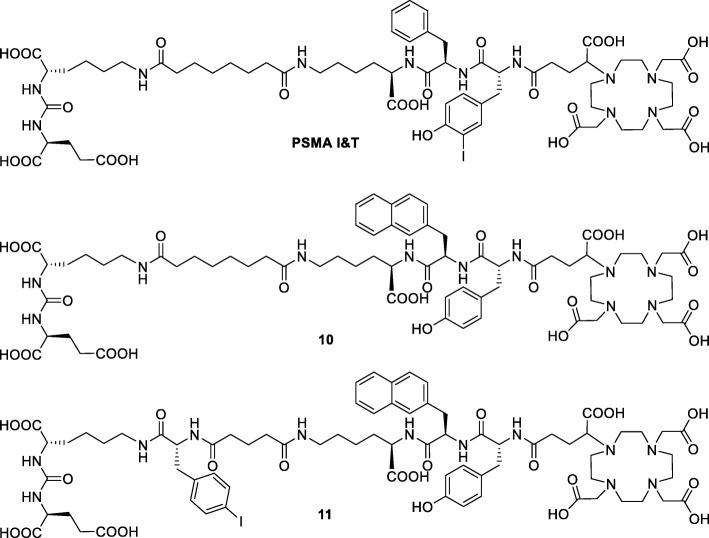
Chemical structures of the DOTAGA-conjugated PSMA inhibitors PSMA I&T, 10, and 11

In the literature, para-halogenated aromatic systems conjugated to the *N*_ɛ_-amino group of KuE seem favorable in terms of PSMA affinity [25] and PSMA inhibitors containing a para-iodo-benzoic acid derivative as albumin binder demonstrated an increased tumor uptake in vivo [26, 27]. In addition, para-halogenated aromatic systems are reported to increase plasma protein binding [28–30], and a reduced tracer uptake in the kidneys was reported for radiopharmaceuticals modified with an albumin-binding entity [31]. Therefore, we designed several derivatives of the known PSMA inhibitor PSMA I&T in terms of structural modification of the spacer unit with lipophilic moieties, and additionally, we introduced a para-halogenated aromatic residue, para-iodo-phenylalanine (I-f), into the linker between the binding motif and the spacer unit. Subsequently, all PSMA ligands were evaluated regarding PSMA affinity, and the most promising structure **6** was further used to develop the PSMA inhibitors **10** and **11** (Fig. 2). These two ligands were radiolabeled with ^68^Ga^III^ and ^177^Lu^III^ and comparatively evaluated with particular respect to internalization in LNCaP cells, lipophilicity, plasma protein binding, and albumin binding. In order to assess the influence of the structural modifications in vivo, both ligands were evaluated in micro-PET imaging and biodistribution studies and compared to PSMA I&T.

## Results and discussion

### Synthesis

The PSMA inhibitors **1**–**9** (Fig. 1) were synthesized in a combined solution and solid-phase synthesis strategy. The acetylated three-amino acid peptides (amino acid spacer in Fig. 1) were assembled using Fmoc-protocol solid-phase peptide synthesis starting from approximately 100 mg TCP resin. Acidic cleavage from the resin and precipitation in diethyl ether yielded the peptides in medium to high yields and purity. Acetyl ester formation at the unprotected side chain hydroxyl group of **8** and **9** necessitated an additional “deprotection step,” which was achieved by alkaline hydrolysis using methanol/water/saturated NaHCO_3_ (2/1/1) in almost quantitative yields. The reaction of the crude peptides with NHS-Sub-(O*t*Bu)KuE(O*t*Bu)_2_ was performed according to a literature procedure [22]. After removal of the solvent and acidic deprotection, the PSMA inhibitors were purified using RP-HPLC.

The DOTAGA-conjugated inhibitor **10** was synthesized from the DOTAGA-y-nal-k peptide and PfpO-Sub-(O*t*Bu)KuE(O*t*Bu)_2_ yielding the PSMA inhibitor after *t*Bu deprotection and HPLC purification. The NHS ester of suberic acid is commercially available; however, the PfpO ester (Sub(OPfp)_2_) was synthesized in 68% yield from affordable reagents resulting in a less hydrolysis-prone building block.

Hydrolytic stability was especially important for the synthesis of PSMA inhibitor **11**. To address plasma proteins, a PSMA inhibitor with a halogenated aromatic moiety (4-iodo-d-phenylalanine; I-f) was developed on the basis of **10**. Substitution of the suberic acid spacer with glutaric acid-(I-f) resulted in DOTAGA-y-nal-k(Glut-(I-f)-KuE) (**11**). For the synthesis of **11**, PfpO-Glut-(I-f)-(O*t*Bu)KuE(O*t*Bu)_2_ was synthesized in solution from (O*t*Bu)KuE(O*t*Bu)_2_, Fmoc-d-iodo-phenylalanine, and the bis-pentafluorophenylester of glutaric acid (Glut(OPfp)_2_) and was purified using RP-HPLC. Glut(OPfp)_2_ was prepared from glutaric acid using pyridine and pentafluorophenol in 87% yield after flash chromatography. Synthesis yields of PSMA inhibitors **1**–**11** are summarized in Additional file 1: Table S1.

### Affinity to PSMA

The effect of the lipophilic (aromatic) modifications in the spacer unit of the acetylated PSMA inhibitors **1**–**9** on the *IC*_*50*_ to PSMA was determined in a competitive binding assay on PSMA-expressing LNCaP cells. (((S)-1-carboxy-5-(4-([13]iodo-benzamido)pentyl)carbamoyl)-l-glutamic acid (^125^I-**IBA**)) in a concentration of 0.2 nM was used as radioligand [22]. The means of three independent measures are summarized in Table 1. The acetylated L-derivative, Ac-YFK(Sub-KuE) (**1**), was included to ensure comparability with the DOTAGA-conjugated compound PSMA I&T [17, 22] and revealed a lower affinity compared to it (15.0 ± 1.3 nM vs. 10.2 ± 3.5 nM, 1 vs. PSMA I&T, respectively).

**Table 1:** Affinities (*IC*_*50*_ values) of the PSMA inhibitors in this study as determined in a competitive binding assay on LNCaP cells (150,000 cells/well, 4 °C, 1 h, c(^**125**^I-**IBA**) = 0.2 nM as the reference ligand). Data are expressed as mean ± SD (*n* = 3). *Data were taken from Ref. [17]

PSMA inhibitor	PSMA inhibitors (amino acid code)	IC_50_
1	Ac-YFK(Sub-KuE)	15.0 ± 1.3
2	Ac-YWK(Sub-KuE)	6.8 ± 3.3
3	Ac-Y-(Benzothienyl-A)K(Sub-KuE)	10.2 ± 4.0
4	Ac-Y-(Biphenyl-A)K(Sub-KuE)	139.4 ± 117.0
5	Ac-Y-1-Nal-K(Sub-KuE)	4.3 ± 0.9
6	Ac-Y-2-Nal-K(Sub-KuE)	3.9 ± 1.7
7	Ac-Y(4-NO_2_-F)K(Sub-KuE)	7.4 ± 0.5
8	Ac-Y(3,5-di-I-Y)K(Sub-KuE)	3.8 ± 0.6
9	Ac-Y(3-CH_3_-Y)K(Sub-KuE)	7.2 ± 0.9
PSMA I&T	DOTAGA-(I-y)fk(Sub-KuE)	10.2 ± 3.5*
^nat^Ga-PSMA I&T	^nat^Ga-DOTAGA-(I-y)fk(Sub-KuE)	9.3 ± 3.3*
^nat^Lu-PSMA I&T	^nat^Lu-DOTAGA-(I-y)fk(Sub-KuE)	7.9 ± 2.4*
10	DOTAGA-y-2-nal-k(Sub-KuE)	8.5 ± 2.5
^nat^Ga-10	^nat^Ga-DOTAGA-y-2-nal-k(Sub-KuE)	9.8 ± 3.2
^nat^Lu-10	^nat^Lu-DOTAGA-y-2-nal-k(Sub-KuE)	2.1 ± 0.8
11	DOTAGA-y-2-nal-k((I-f)-Glut-KuE)	4.6 ± 0.9
^nat^Ga-11	^nat^Ga-DOTAGA-y-2-nal-k((I-f)-Glut-KuE)	9.3 ± 2.9
^nat^Lu-11	^nat^Lu-DOTAGA-y-2-nal-k((I-f)-Glut-KuE)	6.1 ± 1.6

Increased affinity of PSMA inhibitors was reported by interaction with the 20-Å-deep amphipathic funnel-shaped tunnel leading from the enzyme surface to the S_1_ pocket in the active center of PSMA [32]. The bulky substituent biphenylalanine in **4** showed the lowest affinity in this series, most likely due to steric repulsion in this narrow tunnel and suboptimal fit into the arene-binding site [13, 33]. Yet, in a recent investigation, a PSMA inhibitor with a similar biphenyl residue demonstrated the highest K_i_ among the tested set of ligands [34].

Exhibiting similar lipophilicity and steric demand with PSMA I&T [17], iodo-tyrosine-like substituents were investigated with inhibitors **7** (4-nitro-phenylalanine) and **9** (methyl-tyrosine). The affinity of **7** and **9**, as well as the tryptophan-containing inhibitor **2**, and the benzothienylalanine-containing inhibitor **3** was comparable to PSMA I&T. The diiodo-tyrosine-containing inhibitor **8**, as well as **5** (1-naphtylalanine) and **6** (2-naphtylalanine), revealed two- to threefold higher affinities compared to PSMA I&T. Due to synthetic problems caused by the unprotected diiodo-tyrosine side chain of **8** and the good availability of the naphthylalanine derivatives, inhibitor **6** was selected for further PSMA inhibitor development.

Interestingly, the lutetium complexes of PSMA I&T, **10**, and **11** revealed higher affinity to PSMA compared to the respective gallium complexes.

Although crystal structure-based characterization of the active center of PSMA revealed an additional lipophilic binding pocket (S_1_ accessory lipophilic pocket) near the S_1_ site [32, 35], the affinity of ^nat^Ga- and ^nat^Lu-**11** was comparable to metallated **10** being in the low nanomolar range. Thus, substitution of suberic acid in inhibitor **10** by glutaric acid-(iodo-phenylalanine) in inhibitor **11** does not increase the affinity to PSMA as reported previously for PSMA inhibitors with aromatic moieties conjugated to the KuE motif [28].

### Internalization and cell binding

To determine an effect on the cell binding and internalization of radiolabeled **10** and **11** (Additional file 1: Figure S1), LNCaP cells (125,000/well) were incubated with 0.2 nM ^68^Ga or 0.5 nM ^177^Lu-**10** or ^177^Lu-**11**, respectively, for up to 1 h at 37 °C as described in the literature [17, 22]. A 2-(phosphonomethyl)pentane-1,5-dioic acid (PMPA) wash step (10 μm, 10 min, 4 °C) was conducted to differentiate between specifically membrane-bound (< 3% of applied dose) and internalized activity. Nonspecific binding, determined by co-incubation with 10 μm PMPA, was below 1% of the applied dose. To compensate for differences in cell count and viability, the external reference ^125^I-**IBA** was always assayed in parallel, and the binding data in Table 2 are given as percentage of the external reference. As expected from the affinity data, the internalization of ^177^Lu-**10** was comparable to ^177^Lu-PSMA I&T. Interestingly, the cell binding and internalization kinetics revealed a significantly increased cellular uptake of ^68^Ga-**10** in LNCaP cells. An increased internalization was also observed for ^68^Ga- and ^177^Lu-**11** compared to radiolabeled **10** and PSMA I&T. Unlike that reported for radiolabeled DOTAGA-ffk(Sub-KuE) and PSMA I&T [17, 22], the internalization experiments do not correlate with the affinity to PSMA. Thus, the affinity and cell binding data indicate that the interaction of PSMA inhibitors **10** and **11** with PSMA differs from PSMA I&T [17, 22] and was therefore further investigated with regards to lipophilicity and in vivo behavior.

**Table 2:** Cell binding and internalization of ^**68**^Ga/^**177**^Lu-**10** and ^**68**^Ga/^**177**^Lu-**11** compared to the reference ligands ^**68**^Ga/^**177**^Lu-PSMA I&T after 1 h on LNCaP cells (125,000/well, DMEM/F-12 + 5% BSA). Data is expressed as percentage of external reference ^**125**^I-**IBA** and as mean ± SD (*n* = 3)

	Cell binding (% of ^125^I-IBA)	Internalization (% of ^125^I-IBA)
^68^Ga-PSMA I&T	65.0 ± 1.7	59.2 ± 1.7
^177^Lu-PSMA I&T	79.6 ± 1.1	75.5 ± 1.6
^68^Ga-10	112.6 ± 2.1	105.1 ± 2.1
^177^Lu-10	77.3 ± 0.7	71.7 ± 0.7
^68^Ga-11	107.6 ± 1.3	107.0 ± 1.2
^177^Lu-11	119.6 ± 0.6	118.6 ± 0.5

### Lipophilicity, plasma protein, and human albumin binding

For the novel PSMA inhibitors, the partition coefficient between n-octanol and PBS (pH 7.4) was determined using the shake-flask method. The log*P* values (*n* = 6 for each) of the ^68^Ga- and ^177^Lu-labeled inhibitors **10** (− 3.8 ± 0.1 and − 4.1 ± 0.1 for ^68^Ga- and ^177^Lu-**10**, respectively) and **11** (− 3.5 ± 0.1 and − 3.1 ± 0.1 for ^68^Ga- and ^177^Lu-**11**, respectively), designed for optimized lipophilic interaction with lipophilic PSMA pockets, was correspondingly higher compared to the radiolabeled derivative PSMA I&T (− 4.3 ± 0.3 and − 4.1 ± 0.1 for ^68^Ga- and ^177^Lu-PSMA I&T, respectively). However, having the hydrophilic KuE motif and the chelator (DOTAGA) at both ends of the molecule, radiolabeled PSMA inhibitors **10** and **11** were still highly hydrophilic compared to other peptides, such as peptides binding the CXCR-4 receptor (log*P* (^68^Ga-CPCR4–2) = − 2.90 ± 0.08) [36].

High in vivo plasma protein binding increases the plasma half-life of the radiopharmaceutical and therefore might offer beneficiary effects on the tracer distribution (higher uptake into target tissue) but can also lead to increased background activity especially at early time points [27]. In general, drugs binding to plasma proteins with high affinity feature moderate to high lipophilicity, in many cases due to halogenated aromatic groups. To estimate the bioavailability of ^177^Lu-PSMA I&T, ^177^Lu-**10**, and ^177^Lu-**11** in the blood circulation, the extent of plasma protein binding was determined by in vitro incubation in human plasma and subsequent ultracentrifugation. Human albumin binding was determined, applying a modified HPLC method [37]. In accordance with an almost similar lipophilicity of ^177^Lu-PSMA I&T and ^177^Lu-**10**, the plasma protein binding of these PSMA inhibitors was 82% and 81%, respectively. These high values might be explained by the multiple negative charges (carboxylates of KuE and DOTAGA) at both ends of the molecules, being connected over a lipophilic peptide spacer, another structural motif reported to bind plasma proteins [31]. In addition, the intercalation of an additional iodo-phenylalanine residue increased the lipophilicity of ^177^Lu-**11** compared to ^177^Lu-**10**. In consistency with the increased lipophilicity, the iodo-phenyl group insertion resulted in almost quantitative plasma protein binding of 97% for ^177^Lu-**11**. Similar results were obtained for the HSA binding. While ^nat^Lu-PSMA I&T and ^nat^Lu-**10** showed values of 79% and 83% bound to HSA, the para-iodo-phenyl-substituted derivative ^nat^Lu-**11** exhibited 97%. The results indicate that the modification with the halogenated aromatic residue increases in first line the albumin binding, which accounts almost completely for the almost quantitative plasma protein binding of ^nat^Lu-**11** in vitro.

### Biodistribution

**Fig. 3:**
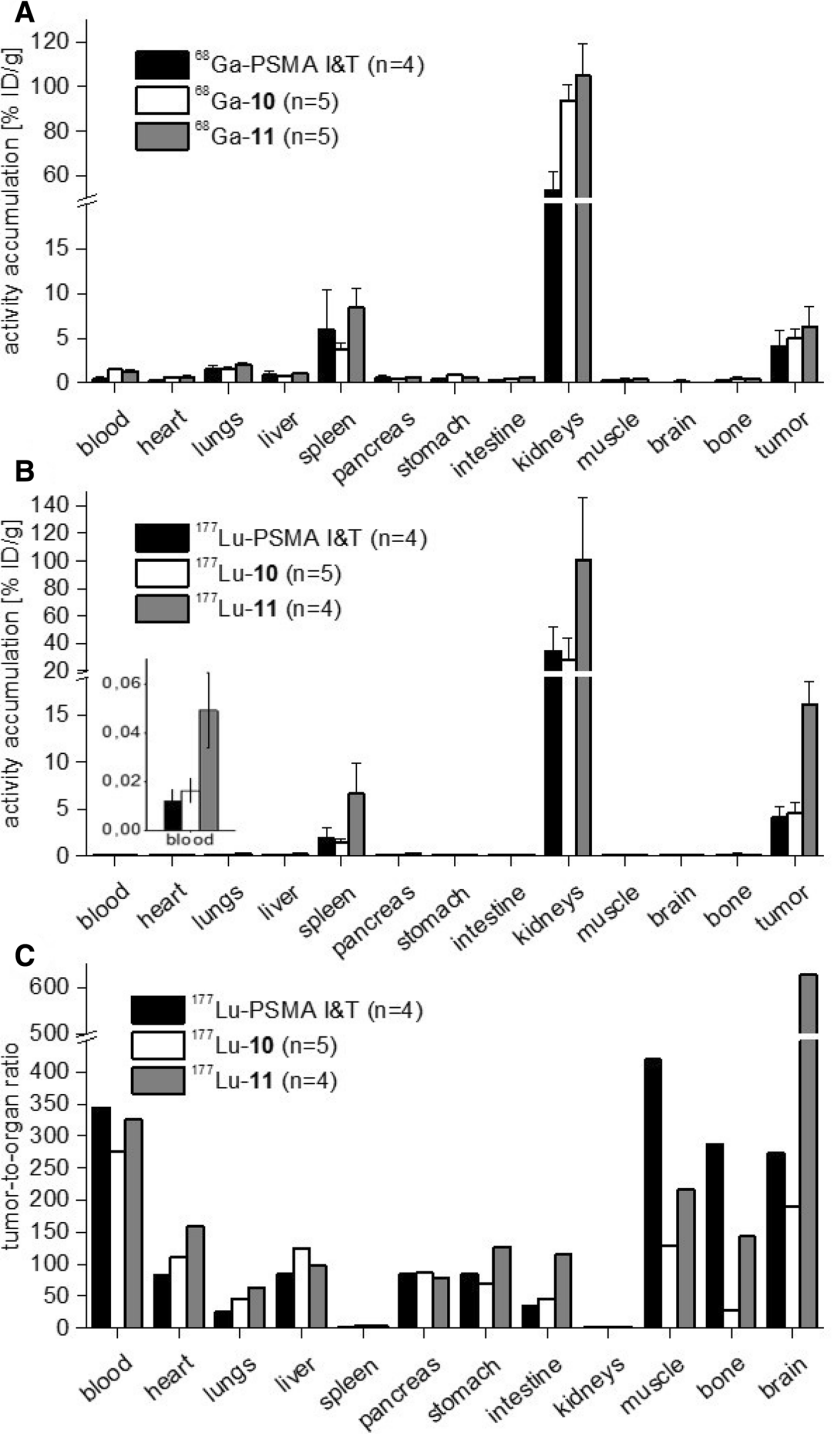
Biodistribution data. **a** Biodistribution of 13.4 ± 0.8 MBq (approximately 0.2 nmol) ^68^Ga-**10** and ^68^Ga-**11** in comparison to ^68^Ga-PSMA I&T at 1 h p.i. in LNCaP tumor-bearing CD-1 nu/nu mice (*n* = 4, respectively). **b** Biodistribution and **c** tumor-to-organ ratios of the respective ^177^Lu-labeled compounds at 24 h p.i. in LNCaP tumor-bearing SCID mice. Data are expressed as mean ± SD (*n* = 4 or 5)

To investigate an influence of the increased internalization of ^68^Ga-**10** and the almost quantitative plasma protein binding of radiolabeled **11** on the in vivo behavior, the biodistribution of ^68^Ga-**10**, ^68^Ga-**11**, and PSMA I&T was determined 1 h after injection in LNCaP tumor-bearing CD-1 nu/nu mice (Fig. 3a). As expected from the highly hydrophilic tracers, their clearance was fast and exclusively via the kidneys. After 1 h, the uptake of ^68^Ga-labeled **11** into the tumor xenograft, the kidneys, and the spleen (all of which are organs with documented PSMA expression [38]) was comparable to ^68^Ga-labeled **10** and PSMA I&T. In consistency with the 97% in vitro plasma protein binding for ^68^Ga-**11**, 1.3 ± 0.1% ID/g was found in the blood after 1 h compared to 0.4 ± 0.2% ID/g for ^68^Ga-PSMA I&T. Interestingly, increased blood retention was also observed for ^68^Ga-**10**, although the HPLC retention, the log*P*, and the plasma protein binding were comparable to ^68^Ga-PSMA I&T. Thus, the increased internalization and elevated blood level of ^68^Ga-**10** and ^68^Ga-**11** compared to ^68^Ga-PSMA I&T merits further investigation at later time points.

With respect to a potential endoradiotherapeutic benefit of the higher blood levels of radiolabeled **10** and **11**, the biodistribution of ^177^Lu-**10** and ^177^Lu-**11** was determined at 24 h p.i. in LNCaP tumor-bearing SCID mice (Fig. 3b). The 24-h p.i. biodistribution of ^177^Lu-**10** revealed washout from all organs and a tumor retention (4.5 ± 1.1% ID/g), which is comparable to ^177^Lu-PSMA I&T (4.1 ± 1.1% ID/g). A fourfold increase of the activity in the LNCaP tumor xenograft at 24 h p.i. was observed for ^177^Lu-**11** with 16.1 ± 2.5% ID/g. The high ^177^Lu-**11** retention in the tumor xenograft is explained by 97% plasma protein binding, which decelerates the excretion and thus increases the uptake in PSMA-specific tissues with time. This hypothesis is supported by the higher blood activity of radiolabeled **11** compared to PSMA I&T at 1 h p.i., which might allow subsequent delivery of the PSMA inhibitor to the tumor over time. However, although the initial blood activity at 1 h p.i. of ^68^Ga-**10** and ^68^Ga-**11** was similar, ^177^Lu-**10** did not show the same effect of increased tumor uptake at 24 h p.i. The data suggest that the initial distribution of both ligands is not significantly influenced by the HSA binding; however, its effect becomes more apparent at later time points. The initial α-phase of the blood clearance is dominated by especially the distribution process for strong and weak plasma protein binding small molecules and leads to a rapid decline of blood pool activity after i.v. injection. However, the difference in the clearance kinetics becomes more visible in the terminal elimination phase. The terminal phase is stronger dominated by elimination processes and influenced by the plasma protein binding properties of the molecule. The ^177^Lu-**11** activity in the spleen (6.6 ± 3.3% ID/g) and the kidneys (100.9 ± 45.4% ID/g) was also higher for ^177^Lu-**11** compared to ^177^Lu-**10**, confirming the effect of higher plasma protein binding on PSMA-expressing tissue uptake, PSMA-specific uptake in these organs. Similar results were observed by other groups, in which the strong albumin-binding ligands exhibited increased tumor accumulation and stronger retention [26, 27, 39]. Kelly et al. suggested that differences in kidney uptake are mediated by albumin binding and that strong binding ligands should display lower renal accumulation. This is, however, in contrary to our observation. Kidney uptake of ^177^Lu-**11** was the highest after 24 h among the tested ligands and resembled more the results of Choy et al. [27].

Although an almost fourfold higher uptake of ^177^Lu-**11** compared to ^177^Lu-**10** and ^177^Lu-PSMA I&T was found at 24 h p.i., the tumor-to-organ ratios (Fig. 3c) of ^177^Lu-PSMA I&T were comparable or higher than that for ^177^Lu-**11**. The high and persistent tumor uptake of ^177^Lu-**11** was caused by a decelerated blood clearance and probably higher metabolic stability due to stronger interactions with albumin and the resulting decreased steric accessibility of metabolizing enzymes towards the PSMA ligand. The increased tumor uptake led to high tumor-to-organ ratios at 24 h p.i., e.g., 326 ± 0.3 (tumor-to-blood) or 143 ± 0.3 (tumor-to-bone). Thus, endoradiotherapeutic application of ^177^Lu-**11** might deliver higher radiation doses to the target tissue compared to ^177^Lu-PSMA I&T and ^177^Lu-**10**. However, the likewise increased blood level and especially the kidney and spleen uptake of ^177^Lu-**11** after 24 h p.i. (Fig. 3b) most likely limits the maximal dose and has to be considered in terms of potential nephro- or hematotoxicity [40]. First-in-man studies are necessary to address the issue if the renal uptake in preclinical studies is a predictive parameter to assess the nephrotoxicity in men. The difference of preclinical results regarding the renal uptake of PSMA I&T (high) and PSMA-617 (low) and the almost identical uptake in clinical studies suggest that the kidney uptake in mice has only limited predictive value [21].

### PET imaging

**Fig. 4:**
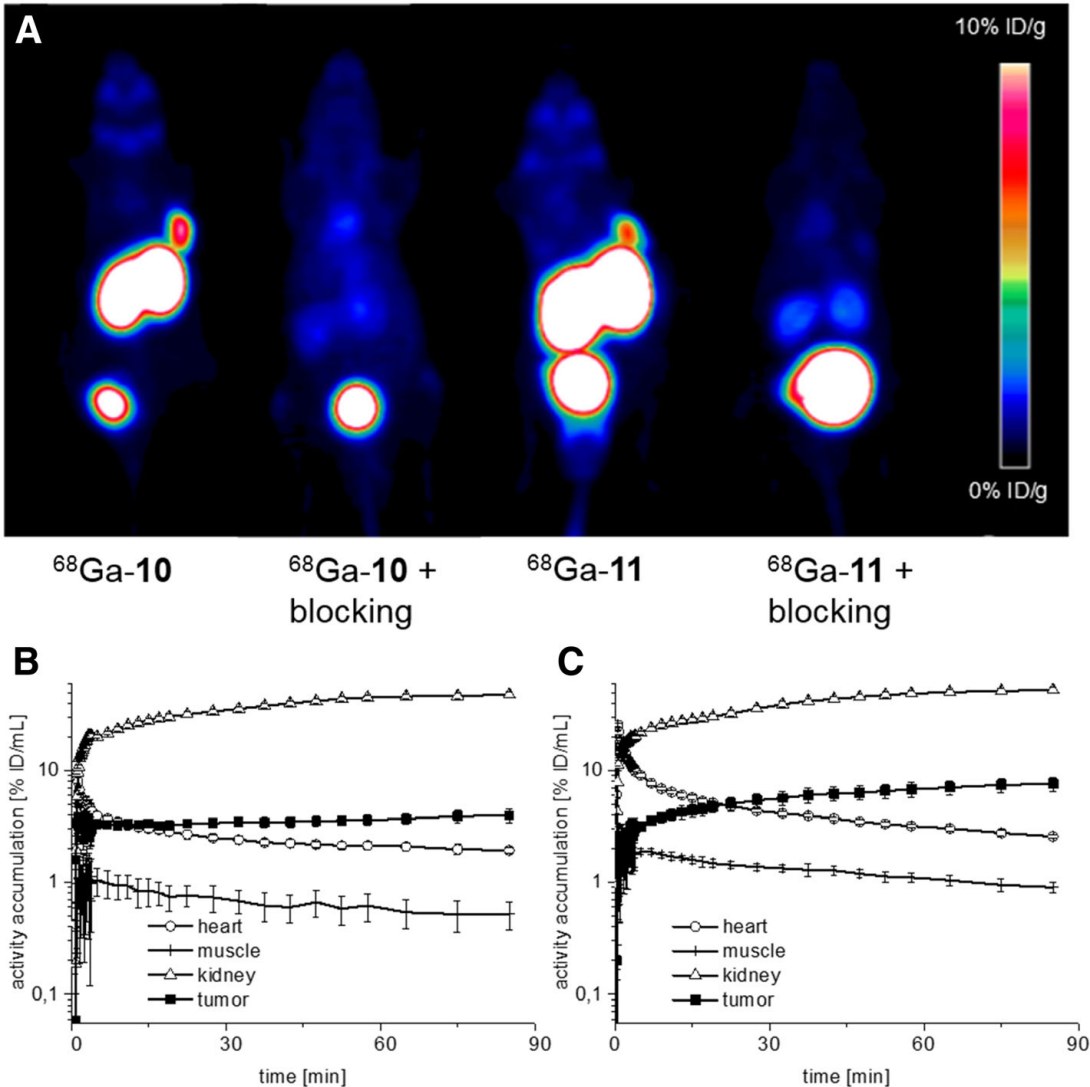
Small-animal positron emission tomography. **a** PET images (MIP at 1 h p.i., 0–10% ID/g) of LNCaP tumor-bearing CD-1 nu/nu mice after injection of ^68^Ga-**10** (0.28 nmol), ^68^Ga-**10** co-injected with PMPA (8 mg/kg), ^68^Ga-**11** (0.23 nmol), and ^68^Ga-**11** co-injected with PMPA (8 mg/kg), respectively (*n*=1). Time-activity curves (logarithmic plot) for the blood pool (heart), muscle, kidney, and tumor as derived from OSEM 3D reconstructed dynamic PET scan for 1.5 h after injection of **b** ^68^Ga-**10** and **c** ^68^Ga-**11** (*n* = 1, respectively)

Figure 4a shows PET images of LNCaP tumor-bearing mice 1 h after injection of ^68^Ga-**10** and ^68^Ga-**11**, respectively. In accordance with the biodistribution data, both tracers were primarily taken up into the tumor and the kidneys, with excretion into the bladder. The PSMA specificity of in vivo binding was confirmed by co-injection of the structurally independent PSMA inhibitor PMPA. In the ^68^Ga-**11** time-activity curves of a 1.5-h observation period (Fig. 4c), the plasma protein bound activity seems to be stronger delivered to PSMA-specific organs compared to ^68^Ga-**10** (Fig. 4b) and therefore leads to an increase in tumor uptake over time, which is consistent with the biodistribution data of ^177^Lu-**11** at 24 h p.i.

## Conclusions

An improved interaction of the PSMA inhibitors with the arene-binding pocket by means of the introduction of bulky and lipophilic bicyclic aromatic side chains in the linker unit resulted in higher affinity and slightly higher internalization in vitro. The delayed excretion kinetics of ^177^Lu-**11** compared to ^177^Lu-**10** and ^177^Lu-PSMA I&T, caused by 97% plasma protein binding of ^177^Lu-**11**, led to a significantly improved tumor uptake at 24 h p.i. However, in the context of the new generation of high plasma protein binding PSMA inhibitors with improved tumor uptake, first-in-man studies have to prove if an increased diagnostic, therapeutic, or theranostic value exists and if high plasma protein binding is the next big step in PSMA drug development.

## Methods

The general experimental procedures are described in detail in Additional file 1.

### Synthetic procedures

The PSMA inhibitors **1**–**9** were synthesized according to previously published methods [22, 41] with minor modifications as described in Additional file 1. For amino acid nomenclature, the one-letter code according to IUPAC-IUB was used.

#### PSMA inhibitor 10

To a solution of 2.0 g (11.5 mmol, 1.0 eq.) suberic acid in 30 mL tetrahydrofuran (THF), 2.8 mL (34.5 mmol, 3.0 eq.) pyridine, 7.1 mL (46.0 mmol, 4.0 eq.) *N,N′*-diisopropylcarbodiimide (DIC) in 15 mL THF, and 8.47 g (46.0 mmol, 4.0 eq.) pentafluorophenol in 15 mL THF were successively added. The progress of the active ester formation was monitored using TLC (ethyl acetate/petroleum ether (55–65 °C) (1/9)). After approximately 2 h at rt, the reaction mixture was filtered, and the solvent was evaporated in vacuo. The crude product was purified via silica gel flash chromatography using an eluent mixture of ethyl acetate/petroleum ether (1/9). Di-pentafluorophenyl suberate (Sub(OPfp)_2_) was obtained as a yellow crystalline solid. Calculated monoisotopic mass for Sub(OPfp)_2_ (C_20_H_12_O_4_F_10_) = 506.1 (product is not detectable using ESI-MS).

To a solution of 400 mg (0.8 mmol, 1.0 eq.) (O*t*Bu)KuE(O*t*Bu)_2_ in 100 mL THF, 274 μL (1.6 mmol, 2.0 eq.) DIPEA was added. This solution was added dropwise (within 30 min) to a solution of 1.6 g (3.2 mmol, 4.0 eq.) Sub(OPfp)_2_. After stirring for an additional 2 h at rt, the reaction mixture was concentrated in vacuo, and the crude product was purified via silica gel flash chromatography using a stepwise gradient of ethyl acetate in petroleum ether (55–65 °C) of 10%, 50%, 90%, and pure ethyl acetate (200 mL each). OPfp-Sub-(O*t*Bu)KuE(O*t*Bu)_2_ was obtained as a yellowish oil in 58% yield. Calculated monoisotopic mass (C_38_H_56_F_5_N_3_O_10_) = 809.4 found *m/z* = 810.6 [M + H]^+^, 832.4 [M + Na]^+^.

Peptide synthesis of y-nal-k and DOTAGA coupling to the deprotected *N*-terminus of the peptide was performed as described previously [22]. DOTAGA-tyrosine-2-naphthylalanine-lysine HPLC (20 to 70% B in 15 min) found *t*_*R*_ = 10.7 min, *K′* = 4.1. Calculated monoisotopic mass (C_47_H_64_N_8_O_14_) = 964.5 found *m/z* = 965.8 [M + H]^+^, 987.8 [M + Na]^+^.

Reaction conditions of the peptide with OPfp-Sub-(O*t*Bu)KuE(O*t*Bu)_2_ were similar as described for the respective NHS ester. DOTAGA-y-2-nal-k(Sub-KuE) (**10**) HPLC (25 to 55% B in 15 min.) found *t*_*R*_ = 12.5 min, *K′* = 6.14. Calculated monoisotopic mass (C_67_H_95_N_11_O_23_) = 1421.7 found *m/z* = 1422.8 [M + H]^+^, 712.1 [M + 2H]^2+^.

#### PSMA inhibitor 11

To a solution of 2.0 g (15.1 mmol, 1.0 eq.) glutaric acid in 15 mL, THF was added 3.7 mL (45.4 mmol, 3.0 eq.) pyridine, 9.5 mL (60.5 mmol, 4.0 eq.) DIC in 10 mL THF, and 11.1 g (60.5 mmol, 4.0 eq.) pentafluorophenol in 10 mL THF. After 2 h, the solvent was removed in vacuo, and the crude dissolved in petrol ether was filtered and purified using silica gel flash chromatography (petrol ether/ethyl acetate = 95/5) yielding 6.1 g (87%) di-pentafluorophenyl glutarate (Glut(OPfp)_2_) as a white crystalline solid. HPLC (10 to 100% B in 15 min) found *t*_*R*_ = 17.5 min, *K′* = 7.75. Calculated monoisotopic mass (C_19_H_36_N_4_O_6_) = 416.3 found *m/z* = 417.1 [M + H]^+^.

A solution of 0.5 g (0.97 mmol, 1.2 eq.) Fmoc-d-4-iodo-phenylalanine, 0.2 g (1.22 mmol, 1.5 eq.) HOAt, 0.2 mL (0.16 g, 1.22 mmol, 1.5 eq.) DIC, and 0.6 mL (0.47 g, 3.65 mmol, 4.5 eq.) DIPEA in 15 mL THF was stirred at rt. for 1 h. After the addition of 395 mg (0.81 mmol, 1.0 eq.) (O*t*Bu)KuE(O*t*Bu)_2_ in 5 mL THF, the reaction mixture was stirred overnight. Water (20 mL) was added and extracted with 25 mL ethyl acetate (3×), followed by 20 mL H_2_O (3×) and 25 mL brine. The organic phase was dried over MgSO_4_, and the solvent was evaporated in vacuo yielding 2.0 g (> 100%) Fmoc-(I-f)-(O*t*Bu)KuE(O*t*Bu)_2_ as a white solid, which was dissolved in 25.0 mL DMF, and 5.0 mL piperidine was added and stirred for 2 h. The crude product was purified using HPLC (58% B isocratic). HPLC (10 to 90%) found *t*_*R*_ = 15.6 min, *K′* = 8.2. Calculated monoisotopic mass (C_33_H_53_IN_4_O_8_) 760.3 found *m/z* = 761.4 [M + H]^+^, 783.4 [M + Na]^+^, 799.4 [M + K]^+^, 593.3 [M-3 *t*Bu + H]^+^, 649.3 [M-2 *t*Bu + H]^+^, 705.3 [M- *t*Bu + H]^+^.

At 0 °C, 270 mg (0.35 mmol, 1.0 eq.) (I-f)-(O*t*Bu)KuE(O*t*Bu)_2_ and 122 μL (0.71 mmol, 2.0 eq.) DIPEA in 20 mL THF were slowly added to 660 mg (1.42 mmol, 4.0 eq.) Glut(OPfp)_2_ dissolved in 10 mL THF. After 2 h at rt., the solvent was removed in vacuo, and the crude product was purified using silica gel flash chromatography (petrol ether/ethyl acetate 10/1 → 1/10) yielding 143 mg (39%) OPfp-Glut-(I-f)-(O*t*Bu)KuE(O*t*Bu)_2_. HPLC (10 to 90% B in 15 min) found *t*_*R*_ = 18.0 min, *K′* = 9.6. Calculated monoisotopic mass (C_44_H_58_F_5_IN_4_O_11_) 1040.3 found *m/z* = 1041.2 [M + H]^+^, 1063.6 [M + Na]^+^.

A solution of 21.6 mg (0.021 mmol, 1.0 eq.) OPfp-Glut-(I-f)-(O*t*Bu)KuE(O*t*Bu)_2_ in DMF was added to 22.3 mg (0.021 mmol, 1.0 eq) DOTAGA-y-nal-k and 14.5 μL (0.104 mmol, 5.0 eq.) TEA and was stirred for 3.5 h. Deprotection of *t*Bu esters was achieved in 1.0 mL TFA within 45 min, and the crude product was precipitated in diethyl ether and purified by HPLC (35% B isocratic), DOTAGA-y-nal-k(Glut-(I-f)-KuE) (**11**) by HPLC (35 to 60%) found *t*_*R*_ = 10.5 min, *K′* = 5.2. Calculated monoisotopic mass (C_73_H_97_IN_12_O_24_) 1652.6 found *m/z* = 1654.9 [M + H]^+^, 1676.0 [M + Na]^+^, 827.7 [M + 2H]^2+^.

### Lipophilicity, plasma protein binding, and human serum albumin binding

For lipophilicity determination, samples of 1–2 MBq ^68^Ga/^177^Lu-**10** or ^68^Ga/^177^Lu-**11** in 500 μL PBS, respectively, were added to 500 μL n-octanol (*n* = 6), mixed gently for 3 min, and centrifuged for 3 min. A 100-μL octanol and a 50-μL PBS sample were quantified using a γ-counter, respectively.

Plasma protein binding was determined in fresh blood samples collected in heparinized tubes, which were centrifuged at 6000 rpm (Biofuge 15, Heraeus Sepatech, Osterode, Germany) to separate plasma from the blood cells. Subsequently, 1–2 MBq ^177^Lu-**10** or ^177^Lu-**11** was added to the fresh plasma, respectively, and was incubated at 37 °C for 15 min. The sample was transferred into a VWR 30K (low protein binding) modified PES ultrafiltration vial and centrifuged at 16000 rpm. Quantification of the activity concentration before and after ultrafiltration was performed in a γ-counter. The data was corrected for non-specific association to the membrane (ultrafiltration of tracer sample in PBS).

HSA binding experiments were performed according to a previously reported method using a binary gradient HPLC system connected to a Chiralpak HSA (5 μm, 50 × 3 mm) analytical column connected to a Chiralpak HSA (5 μm, 10 × 3 mm) guard cartridge (Daicel Chemical Industries) purchased from Chiral Technologies Europe (Illkirch, France) with minor modifications [37]. Mobile phase A was an ammonium acetate buffer (50 mM, pH 6.9), mobile phase B was 2-propanol, the flow rate was 0.5 mL/min, and equilibration with 0% B for 3 min was followed by a gradient of 0% B to 20% B to the end of each run within 45 min. To check the column performance and to conduct the non-linear regression, the HSA column was calibrated each day with nine reference substances. Afterward, the PSMA inhibitors with unknown HSA binding were measured. The retention times and factors together with correlation curve are found in Additional file 1.

### In vitro evaluation

Experimental details for the determination of the affinity to PSMA (*IC*_*50*_), the cell binding and internalization kinetics, and the specificity of binding in vitro were described previously [17, 22] and are summarized in Additional file 1.

### Biodistribution and PET imaging

All animal experiments were conducted in accordance with the German Animal Welfare Act (Deutsches Tierschutzgesetz, approval #55.2-1-54-2532-71-13). Tumor induction and experimental details were described previously [17, 22] and are summarized in Additional file 1.

## Additional file

Additional file 1: Supporting information contains the synthetic yields and exemplary illustrations of cell binding studies and internalization kinetics of the investigated PSMA inhibitors. Further, the general experimental procedures are described and contain additional information regarding the synthetic procedure, metal complexation, and radiolabeling. The procedure for affinity determination as well as the procedures for the determination of binding specificity and internalization, albumin binding, biodistribution, and PET-imaging are included. (DOCX 109 kb)

## Abbreviations

DIC: *N,N′*-Diisopropylcarbodiimide
DOTAGA: 1,4,7,10-Tetraazacyclododececane,1-(glutaric acid)-4,7,10-triacetic acid
HSA: Human serum albumin
PET: Positron-emission tomography
Pfp: Pentafluorophenyl
PMPA: 2-(Phosphonomethyl) pentane-1,5-dioic acid
PSMA: Prostate-specific membrane antigen
TCP: Tritylchloride polystyrene
THF: Tetrahydrofuran
