# Supplementary material for: Synthesis and in vitro and in vivo evaluation of urea-based PSMA inhibitors with increased lipophilicity
Source: EJNMMI Res. 2018 Aug 22;8:84. doi: 10.1186/s13550-018-0440-2 (PMC6104465; doi:10.1186/s13550-018-0440-2)
Supplement: Supplementary file 1 — Supporting information contains the synthetic yields and exemplary illustrations of cell binding studies and internalization kinetics of the investigated PSMA inhibitors. Further, the general experimental procedures are described and contain additional information regarding the synthetic procedure, metal complexation, and radiolabeling. The procedure for affinity determination as well as the procedures for the determination of binding specificity and internalization, albumin binding, biodistribution, and PET-imaging are included. (DOCX 109 kb) [file 13550_2018_440_MOESM1_ESM.docx]

# Additional file 1

## RESULTS

The synthesis yields for PSMA inhibitors **1** – **11** is summarized in Table S1.

Table S1 Synthesis yields of the amino acid spacers (acetylated or DOTAGA-conjugated) and the respective PSMA inhibitors 1-11.

| PSMA inhibitor | Peptide synthesis yields  (amino acid spacer) | Yield of inhibitor syntheses (from the respective peptides) |
| --- | --- | --- |
| 1 | 30 mg | 0.76 mg (6%) |
| 2 | 34 mg | 1.3 mg (10%) |
| 3 | 31 mg | 0.44 mg (3%) |
| 4 | 33 mg | 1.4 mg (11%) |
| 5 | 37 mg | 1.3 mg (9%) |
| 6 | 25 mg | 1.7 mg (11%) |
| 7 | 35 mg | 1.7 mg (9%) |
| 8 | 61 mg | 1.1 mg (3%) |
| 9 | 58 mg | 1.43 mg (8%) |
| 10 | 158 mg | 0.94 mg (2%) |
| 11 | 158 mg | 1.71 mg (5%) |

The cell binding and internalization kinetics of ^177^Lu-**10** and -**11** are exemplarily shown in Suppl. Figure 1.


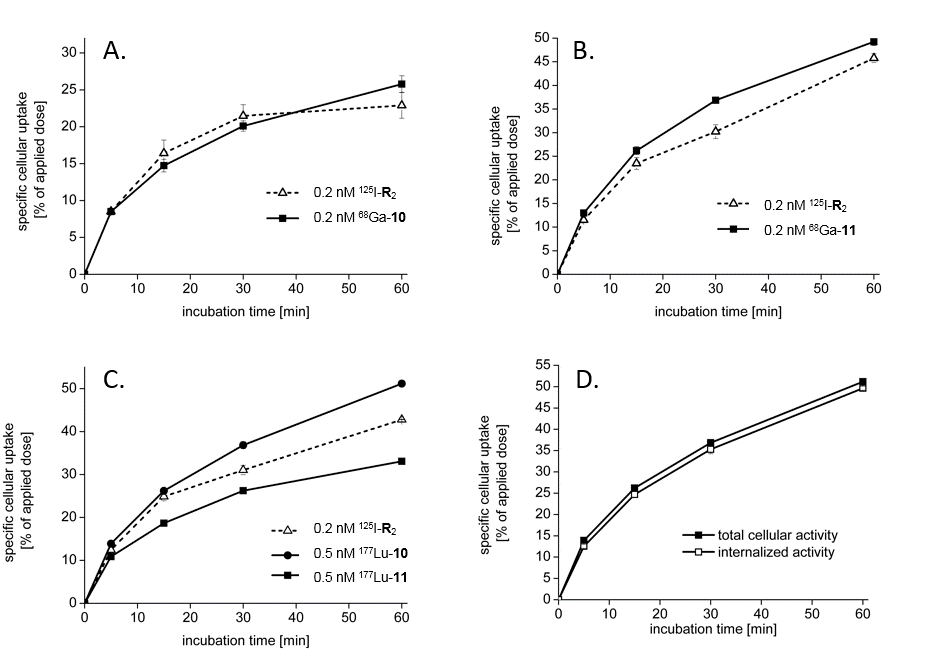


Figure S1 A. Cellular uptake kinetics of A. ^68^Ga-10 B. ^68^Ga-11 C. ^177^Lu-10 and ^177^Lu-11 in comparison to ^125^I-R_2_ (dashed curve), respectively. D. Cell binding and internalized activity of ^177^Lu-12 in LNCaP cells (37 °C, DMEM/F-12 + 5% BSA). The total cellular activity was corrected for non-specific binding (10 μM PMPA). All data are expressed as mean ± SD (n = 3).

## EXPERIMENTAL PROCEDURES

All solvents and chemicals were purchased from SigmaAldrich, CLN or VWR International (Ismaning, Germany) and used without further purification. Fmoc-protected amino acids were obtained from Iris Biotech GmbH (Marktredwitz, Germany), Novabiochem (Merck Millipore, Darmstadt, Germany), BaChem (Bubendorf, Switzerland) and PolyPeptide Laboratories (Straßburg, France). Pipettes and cell culture flasks (175 cm^2^, Cellstar) from greiner bio-one (Frickenhausen, Germany), trypsin/ethylenediaminetetraacetic acid solution (0.05%/0.02% (v/v)) in phosphate buffered saline (PBS) from Dulbecco (without Ca/Mg), Hanks‘ Buffered Salt Solution (0.35 g/L NaHCO_3_) from Biochrom AG (Berlin, Germany), and Dulbecco’s Modified Eagle Medium/F-12 nutrient mixture (DMEM/F-12 (1/1) + GlutaMAX^TM^-I) from Gibco/Life Technologies (California, USA) supplemented with 10% fetal calf serum (FBS Superior, Biochrom AG) were used. The 24-well plates were purchased from Cellstar, greiner bio-one.

Analytical HPLC was performed using a Sykam Gradient HPLC system as described previously [1]. In brief, a Nucleosil 100-5 C18 column (125 x 4.6 mm, Chromatographie Service GmbH) was eluted using water (0.1% trifluoroacetic acid (TFA), v/v) and acetonitrile (0.1% TFA, v/v) at a constant flow of 1 mL/min. Retention times (*t_R_*) and capacity factors (*K’*) are listed in the text.

For preparative HPLC purifications a Sykam S2000 gradient HPLC system equipped with a Multospher 100 RP 18-5μ column (250 x 20 mm) was used (flow rate 10 mL/min). Products were characterized using a Varian 500-MS IT mass spectrometer with ion trap detection. For activity measurement an automated γ-counter (1480 WIZARDTM 3II) was used.

### Synthetic procedures

**Resin loading:** To a suspension of 1.0 eq. dry tritylchloride polystyrene resin (TCP-resin, loading according to the manufacturer: 1.95 mmol/g) in dichloromethane (DCM) was added 2.5 eq. Fmoc-l-Lys(Boc)-OH and 3.75 eq. diisopylethylamine (DIPEA) and stirred for 1.5 h at room temperature. After the addition of 2 mL methanol stirring was continued for another 20 min. The resin was filtered, washed with DCM, *N*,*N*-dimethylsulfoxide (DMF) and methanol, and dried in a desiccator. The actual resin loading was calculated from the weight difference.

**Standard Fmoc-solid-phase peptide synthesis (SPPS):** After swelling in *N*-methyl-pyrrolidone (NMP) (30 min), Fmoc-deprotection was accomplished by incubation in 20% (v/v) piperidine in NMP (10 mL/g resin; 5 and 15 min, respectively). After washing with NMP (8 ×), 1.5 eq. of the respective amino acid, 1.5 eq. 1-hydroxybenzotriazole (HOBt), 1.5 eq. *O*-(1H-benzotriazol-1-yl)-*N*,*N*,*N*’,*N*’-tetramethyluronium-tetrafluoroborate (TBTU), and 4.5 eq. DIPEA were added to the resin and were shaken for 1 h. The coupling solution was filtered off, the resin was washed with NMP (6 ×) and the procedure was repeated for the third amino acid (phenylalanine or tyrosine).

**Peptide acetylation:** After Fmoc-deprotection, the peptide was incubated in NMP/acetic anhydride/DIPEA (85/10/5) for 15 min. The resin was washed with NMP (8 ×) and DCM (3 ×), before the peptide was cleaved from the resin using TFA/TIPS/water (95/2.5/2.5). The solvent was evaporated and the crude product precipitated in diethylether.

Acetyl-tyrosine-phenylalanine-lysine-acetyl HPLC (20 to 70% B in 15 min): *t_R_*= 8.2 min *K‘*= 3.9; Calculated monoisotopic mass for (C_26_H_34_N_4_O_6_) = 498.25 found: *m/z*= 499.3 [M+H]^+^, 521.3 [M+Na]^+^.

Acetyl-tyrosine-tryptophane-lysine HPLC (20 to 70% B in 15 min): *t_R_*= 8.9 min *K‘*= 3.6; Calculated monoisotopic mass for (C_28_H_35_N_5_O_6_) = 537.26 found: *m/z*= 538.3 [M+H]^+^.

Acetyl-tyrosine-benzothienylalanine-lysine HPLC (20 to 70% B in 15 min): *t_R_*= 10.6 min *K‘*= 4.5; Calculated monoisotopic mass for (C_28_H_34_N_4_O_6_S) = 554.22 found: *m/z*= 555.3 [M+H]^+^, 577.2 [M+Na]^+^.

Acetyl-tyrosine-biphenylalanine-lysine HPLC (20 to 70% B in 15 min): *t_R_*= 12.2 min *K‘*= 5.3; Calculated monoisotopic mass for (C_32_H_38_N_4_O_6_) = 574.28 found: *m/z*= 575.3 [M+H]^+^, 597.3 [M+Na]^+^.

Acetyl-tyrosine-1-naphthylalanine-lysine HPLC (20 to 70% B in 15 min): *t_R_*= 10.3 min *K‘*= 4.3; Calculated monoisotopic mass for (C_30_H_36_N_4_O_6_) = 548.26 found: *m/z*= 549.3 [M+H]^+^, 571.3 [M+Na]^+^.

Acetyl-tyrosine-2-naphthylalanine-lysine HPLC (20 to 70% B in 15 min): *t_R_*= 10.3 min *K‘*= 4.3; Calculated monoisotopic mass for (C_30_H_36_N_4_O_6_) = 548.26 found: *m/z*= 549.3 [M+H]^+^, 571.2 [M+Na]^+^.

Acetyl-tyrosine-4-nitrophenylalanine-lysine HPLC (20 to 70% B in 15 min): *t_R_*= 8.8 min *K‘*= 3.5; Calculated monoisotopic mass for (C_26_H_33_N_5_O_8_) = 543.23 found: *m/z*= 544.3 [M+H]^+^, 566.2 [M+Na]^+^.

Acetyl-tyrosine-3,5-diiodotyrosine-lysine HPLC (20 to 70% B in 15 min): *t_R_*= 10.0 min *K‘*= 4.2; Calculated monoisotopic mass for (C_26_H_32_I_2_N_4_O_7_) = 766.04 found: *m/z*= 767.1 [M+H]^+^, 831.3 [M+Na]^+^.

Acetyl-tyrosine-3-methyltyrosine-lysine HPLC (20 to 70% B in 15 min): *t_R_*= 7.5 min *K‘*= 2.8; Calculated monoisotopic mass for (C_27_H_36_N_4_O_7_) = 528.26 found: *m/z*= 529.3 [M+H]^+^, 551.3 [M+Na]^+^.

**PSMA inhibitors 1 ‑ 9:** To a solution of 1.1 eq. disuccinimidyl suberate in DMF was added 1.0 eq. (O*t*Bu)KuE(O*t*Bu)_2_ (synthesized as described [1]) and 5.0 eq. triethylamine (TEA) and stirred for 2 h at room temperature. After extraction in ethyl acetate/water, the organic layer was evaporated in vacuo. The crude product was reacted with the respective peptides in presence of 15 eq. TEA for 3 h, the solvent was evaporated. After tBu-deprotection (pure TFA for 45 min), the crude products were precipitated in diethylether and purified using HPLC. For usage in all further experiments 2 mm solutions of the respective PSMA inhibitors were prepared in tracepure water.

Ac-YFK(Sub-KuE) (**1**) HPLC (30 to 45% B in 15 min): *t_R_*= 7.4 min *K‘*= 4.3; Calculated monoisotopic mass for (C_46_H_65_N_7_O_15_) = 955.45 found: *m/z*= 956.5 [M+H]^+^.

Ac-YWK(Sub-KuE) (**2**) HPLC (25 to 45% B in 15 min): *t_R_* = 15.3 min *K‘*= 9.9; Calculated monoisotopic mass for (C_48_H_66_N_8_O_15_) = 994.46 found: *m/z*= 995.3 [M+H]^+^, 1,017.3 [M+Na]^+^.

Ac-Y-(Benzothienyl-A)K(Sub-KuE) (**3**) HPLC (35 to 45% B in 15 min): *t_R_*= 13.2 min *K‘*= 9.2; Calculated monoisotopic mass for (C_48_H_65_N_7_O_15_S) = 1,011.43 found: *m/z*= 1,012.4 [M+H]^+^, 1,034.3 [M+Na]^+^.

Ac-Y-(Biphenyl-A)K(Sub-KuE) (**4**) HPLC (25 to 55% B in 15 min): *t_R_*= 15.4 min *K‘*= 10.8; Calculated monoisotopic mass for (C_52_H_69_N_7_O_15_) = 1,031.49 found: *m/z*= 1,032.3 [M+H]^+^,1,054.2 [M+Na]^+^.

Ac-Y-1-Nal-K(Sub-KuE) (**5**) HPLC (35 to 45% B in 15 min): *t_R_*= 13.6 min *K‘*= 8.7; Calculated monoisotopic mass for (C_50_H_67_N_7_O_15_) = 1,005.47 found: *m/z*= 1,006.4 [M+H]^+^, 1,028.3 [M+Na]^+^.

Ac-Y-2-Nal-K(Sub-KuE) (**6**) HPLC (35 to 45% B in 15 min): *t_R_*= 13.7 min *K‘*= 8.8; Calculated monoisotopic mass for (C_50_H_67_N_7_O_15_) = 1,005.47 found: *m/z*= 1,006.4 [M+H]^+^,1,028.2 [M+Na]^+^.

Ac-Y(4-NO_2_-F)K(Sub-KuE) (**7**) HPLC (25 to 55% B in 15 min): *t_R_*= 11.8 min *K‘*= 6.9; Calculated monoisotopic mass for (C_50_H_67_N_7_O_15_) = 1,000.44 found: *m/z*= 1,001.6 [M+H]^+^, 1,023.8 [M+Na]^+^.

Ac-Y(3,5-di-I-Y)K(Sub-KuE) (**8**) HPLC (25 to 55% B in 15 min): *t_R_*= 14.8 min *K‘*= 8.9; Calculated monoisotopic mass for (C_50_H_67_N_7_O_15_) = 1,223.24 found: *m/z* = 1,224.6 [M+H]^+^, 1,246.5 [M+Na]^+^.

Ac-Y(3-CH_3_-Y)K(Sub-KuE) (**9**) HPLC (25 to 55% B in 15 min): *t_R_*= 10.1 min *K‘*= 5.7; Calculated monoisotopic mass for (C_50_H_67_N_7_O_15_) = 985.46 found: *m/z*= 986.8 [M+H]^+^, 1,008.9 [M+Na]^+^.

**Metal complexation:** Complexation of cold gallium with PSMA inhibitor **10** or **11** was performed as described previously by heating an equimolar amount of peptide with a 2 mm Ga(NO_3_)_3_ solution to 95 °C for 30 min. The corresponding ^nat^Lu^III^-complexes were prepared from a 2 mm aqueous solution of the respective PSMA inhibitor with a 2.5 molar excess of LuCl_3_ (20 mm solution), heated to 95 °C for 30 min. Quantitative complex formation was determined by HPLC and MS analysis [1].

^nat^Ga-**10** HPLC (25 to 55% B in 15 min): *t_R_* = 10.8 min; *K' =*8.0. Calculated monoisotopic mass (C_67_H_92_N_11_O_23_Ga) = 1,487.6/1,489.6 found: *m/z* = 1,489.0/1,490.9 [M+H]^+^, 1510.9/1512.9 [M+Na]^+^, 746.1 [M+2H]^+^.

^nat^Lu-**10** HPLC (25 to 55% B in 15 min): *t_R_* = 11.6 min; *K' =*8.7. Calculated monoisotopic mass (C_67_H_92_N_11_O_23_Lu): 1,593.6 found: *m/z* = 1,595.0 [M+H]^+^, 1,616.8 [M+Na]^+^, 798.2 [M+2H]^+^.

^nat^Ga-**11** HPLC (25 to 55% B in 15 min): *t_R_* = 11.8 min; *K' =*5.6. Calculated monoisotopic mass (C_73_H_94_IN_12_O_24_Ga): 1,718.5, 1,720.5 found: *m/z* = 1,719.4/1,721.5 [M+H]^+^, 1,740.5/1,742.6 [M+Na]^+^, 861.4 [M+2H]^+^.

^nat^Lu-**11** HPLC (25 to 55% B in 15 min): *t_R_* = 10.6 min; *K' =*4.9. Calculated monoisotopic mass (C_73_H_94_IN_12_O_24_Lu): 1,825.5 found: *m/z* = 1,826.5 [M+H]^+^, 1,848.5 [M+Na]^+^, 913.8 [M+2H]^+^.

### Radiolabeling

The precursor synthesis and subsequent ^125^I-radioiodination of ((S)-1-carboxy-5-(4-(-^125^I-iodo-benzamido)pentyl)carbamoyl)-l-glutamic acid (([^125^I]I-BA)KuE) was described previously [1]. Labeling of **10** and **11** with ^68^Ga^III^ and ^177^Lu^III^ was either performed manually or fully automated as described for other DOTAGA-conjugated PSMA inhibitors in previous reports [1].

### Affinity determination (IC_50_)

LNCaP cells (CLS: 300265) were cultured in Dulbecco modified Eagle medium/Nutrition Mixture F-12 with Glutamax-I (1:1) supplemented with 10% fetal calf serum at 37 °C in a 5% CO_2_/humidified air atmosphere. Cells were harvested using Trypsin/EDTA (0.05% and 0.02%) in PBS, centrifuged and resuspended with culture medium. For cell counting, a Countesse automated cell counter (Invitrogen, Carlsbad, USA) was used.

The PSMA affinity was determined as described previously [1, 2]. In brief, 150,000 LNCaP cells were seeded into 24-well plates one day prior to the experiment. In a competitive binding experiment, 0.2 nm ([^125^I]I-BA)KuE and increasing concentrations of the respective PSMA inhibitor (10^‑5^ to 10^‑11^ m) were incubated for 1 h (4 °C, HBSS supplemented with 1% BSA). After removal of the supernatant, the cells were washed once with HBSS and subsequently lysed with 1 n NaOH. Both fractions were quantified in a γ-counter. Using the Graphpad Prism software, binding curves were plotted and *IC_50_* values were extracted. Three independent determinations were performed in triplicate for each compound.

### Binding specificity and internalization

Internalization kinetics were determined as described previously [1, 2]. In brief, 125,000 LNCaP cells were seeded into poly-l-lysine coated 24-well plates 24 h prior to the experiment. After incubation of 0.2 nm 68Ga/177Lu-10 or -11 at 37 °C for up to 1 h, the supernatant was removed and combined with 250 µL PBS using for rinsing the wells. The cells were subsequently subjected a PMPA wash-step (10 µm in PBS, 10 min, 4 °C), which was combined with another 250 µL PBS using for rinsing the wells. Finally, the cells were lysed using 1 n NaOH and the wells were washed with PBS. All combined fractions were counted in a γ-counter.

### Human Serum Albumin Binding

HSA binding experiments were performed as previously described [3]. Briefly, the mobile phase consisted of a binary gradient system with a constant total flow rate of 0.5 mL/min. Mobile phase A was a 50 mm pH 6.9 NH_4_OAc-solution, mobile phase B was 2-Propanol (RP-HPLC grade, VWR, Germany). The gradient of mobile phase A was 100% from 0 to 3 min and from 3 min to the end of each run mobile phase B was set 20%. At each experimental day, the column was calibrated with nine reference substances to confirm the performance and to establish the non-linear regression. PSMA inhibitors were dissolved in a 0.5 mg/mL concentration in a mixture of 2-Propanol and NH_4_OAc-buffer (50 mm pH 6.9) (1/1; *v/v*). For each run, 10 µL of the solution containing the inhibitor was injected into the RP-HPLC system and the retention time measured. The literature HSA binding [%] was obtained from Valko et. al. or Yamazaki et al. [3, 4]. Non-linear regression was established with OriginPro 2016G.

Table S2. Reference substances used for the calibration of the HSA-column. The retention time is shown exemplary for a conducted experiment; *t_R_* retention time; Lit. HSA literature value of human serum albumin binding in [%]; Log K HAS logarithmic K of human serum albumin binding

| Reference | *t_R_* | Log *t_R_* | Lit. HSA% | Log *K* HSA |
| --- | --- | --- | --- | --- |
| p-benzylalcohol | 2.40 | 0.38 | 13.15 | -0.82 |
| Aniline | 2.72 | 0.43 | 14.06 | -0.79 |
| Phenol | 3.28 | 0.52 | 20.69 | -0.59 |
| Benzoic Acid | 4.08 | 0.61 | 34.27 | -0.29 |
| Carbamazepine | 4.15 | 0.62 | 75.00 | 0.46 |
| p-nitrophenol | 5.62 | 0.75 | 77.65 | 0.52 |
| Estradiol | 8.15 | 0.91 | 94.81 | 1.19 |
| Probenecid | 8.84 | 0.95 | 95.00 | 1.20 |
| Glibenclamide | 29.18 | 1.47 | 99.00 | 1.69 |

Figure S2. Exemplary correlation of determination of the nine reference substances in OriginPro 2016G.

### Biodistribution

All animal experiments were conducted in accordance with German Animal Welfare Act (Deutsches Tierschutzgesetz, approval #55.2-1-54-2532-71-13). To induce tumor growth, LNCaP cells (10^7^ cells/100 µL) were suspended 1/1 in medium and Matrigel (BD Biosciences, Germany) and were inoculated subcutaneously onto the right shoulder of male 6–8 weeks old CD-1 nu/nu or SCID mice (Charles River Laboratories). After 2–4 weeks, tumors had reached 4–8 mm in diameter, and the animals were used for experiments. Under isoflurane anesthesia 12‑14 MBq (app. 0.2 nmol) ^68^Ga-**10** or -**11** were injected into the tail vein of LNCaP tumor–bearing CD-1 mice, respectively. At 1 h after injection of the ^68^Ga-tracers the mice were sacrificed, and the organ distribution in weighed tissue samples was quantified in a γ-counter. The biodistribution of 2.5 – 3.1 MBq ^177^Lu-**10** or -**11** (0.1 – 0.2 nmol) was assessed at 24 h p.i. in LNCaP-tumor bearing SCID mice, respectively.

### PET imaging

Imaging studies were performed on a Siemens Inveon small-animal PET scanner. For data analysis the Inveon Research Workplace software was used. Under isoflurane anesthesia, 14–16 MBq (app. 0.2 nmol) ^68^Ga-**10** or -**11** were injected into the tail vein of LNCaP-tumor bearing mice, respectively. For blocking experiments the tracers were co-injected with 8 mg/kg PMPA in PBS. Static images were recorded at 1 h after injection with an acquisition time of 15 min. Dynamic imaging was acquired under isoflurane anesthesia for 90 min after tracer injection. Images were reconstructed using 3-dimensional ordered-subsets expectation maximum algorithm without scanner and attenuation correction.

1. Weineisen, M., et al., *Synthesis and preclinical evaluation of DOTAGA-conjugated PSMA ligands for functional imaging and endoradiotherapy of prostate cancer.* EJNMMI research, 2014. **4**(1): p. 63.

2. Weineisen, M., et al., *68Ga- and 177Lu-Labeled PSMA I&T: Optimization of a PSMA-Targeted Theranostic Concept and First Proof-of-Concept Human Studies.* Journal of Nuclear Medicine, 2015. **56**(8): p. 1169-1176.

3. Valko, K., et al., *Fast gradient HPLC method to determine compounds binding to human serum albumin. Relationships with octanol/water and immobilized artificial membrane lipophilicity.* Journal of pharmaceutical sciences, 2003. **92**(11): p. 2236-2248.

4. Yamazaki, K. and M. Kanaoka, *Computational prediction of the plasma protein‐binding percent of diverse pharmaceutical compounds.* Journal of pharmaceutical sciences, 2004. **93**(6): p. 1480-1494.
